# Scaffold-free, Human Mesenchymal Stem Cell-Based Tissue Engineered Blood Vessels

**DOI:** 10.1038/srep15116

**Published:** 2015-10-12

**Authors:** Youngmee Jung, HaYeun Ji, Zaozao Chen, Hon Fai Chan, Leigh Atchison, Bruce Klitzman, George Truskey, Kam W. Leong

**Affiliations:** 1 Center for Biomaterials, Biomedical Research Institute, Korea Institute of Science and Technology, Seoul 130-650, Korea; 2 Department of Biomedical Engineering, Duke University, Durham, NC 27708, USA; 3 Department of Biomedical Engineering, Columbia University, New York, NY 10027, USA

## Abstract

Tissue-engineered blood vessels (TEBV) can serve as vascular grafts and may also play an important role in the development of organs-on-a-chip. Most TEBV construction involves scaffolding with biomaterials such as collagen gel or electrospun fibrous mesh. Hypothesizing that a scaffold-free TEBV may be advantageous, we constructed a tubular structure (1 mm i.d.) from aligned human mesenchymal cell sheets (hMSC) as the wall and human endothelial progenitor cell (hEPC) coating as the lumen. The burst pressure of the scaffold-free TEBV was above 200 mmHg after three weeks of sequential culture in a rotating wall bioreactor and perfusion at 6.8 dynes/cm^2^. The interwoven organization of the cell layers and extensive extracellular matrix (ECM) formation of the hMSC-based TEBV resembled that of native blood vessels. The TEBV exhibited flow-mediated vasodilation, vasoconstriction after exposure to 1 μM phenylephrine and released nitric oxide in a manner similar to that of porcine femoral vein. HL-60 cells attached to the TEBV lumen after TNF-α activation to suggest a functional endothelium. This study demonstrates the potential of a hEPC endothelialized hMSC-based TEBV for drug screening.

Drug screening and toxicity studies are crucial steps in the drug development process^1^,^2^. Although any drug candidate has to pass extensive *in vitro* cell-based assays and *in vivo* animal studies before reaching clinical trials, many still fail to progress to commercialization, with an attrition rate approaching 80% of drugs entering a Phase I clinical trial^3^. One drawback of most pre-clinical studies is the failure to recapitulate the human physiological conditions. *In vitro* cell-based assays lack the complex nature of the three-dimensional microenvironment to effectively model tissues or organs^4^,^5^. The responses of non-primate animal studies on the other hand cannot be extrapolated to humans due to cross-species discrepancy. In addition, the time and resources required to construct an animal model appropriate for a specific pathophysiological behavior through surgery or transgenics further incentivize a different approach to advance drug development. An emerging trend is to create an *in vitro* microphysiological system comprising tissue-engineered constructs generated from human cells^6^,^7^,^8^.

One essential and often neglected tissue component of an *in vitro* microphysiological system for drug screening is the blood vessel^9^. Current organ-on-a-chip models connect one tissue compartment to another via plastic channels. However, *in vivo* a drug is exposed to a functional endothelium. Drug-induced vascular injury in animals often confounds the toxicological evaluation in the development of new drugs because of mismatches with humans^10^,^11^. In addition, a functional vasculature is crucial for proper gas and nutrient transport to the surrounding tissues. The failure of vascular function can lead to various diseases, to which many drugs under development are targeting^12^. Thus, the development of a functional artificial blood vessel mimicking the native human vessel is important for drug screening as well as for the construction of an integrated microphysiological system.

The vast majority of tissue-engineered blood vessel (TEBV) development has relied on the use of scaffolds such as synthetic polymers and hydrogels. Despite extensive research, a TEBV that exhibits satisfying functional, biocompatible and mechanical properties has proven difficult to achieve^13^,^14^. L’Heureux *et al.* and Niklason *et al.* showed separately that a TEBV consisting of only cells and extracellular matrices without any synthetic or exogenous materials could exhibit the desirable functional characteristics^15^,^16^. Furthermore, L’Heureux *et al.* validated the use of cell sheets to produce a functional TEBV with smooth muscle cells (SMCs) and endothelial cells (ECs)^17^. Therefore, a scaffold-free TEBV is attractive; the issue is what would be convenient and practical cell sources.

Human mesenchymal stem cells (hMSCs) are an attractive cell source for the medial layer of TEBVs based on their unique antithrombogenic properties, immune response modulation abilities, and multipotency for differentiation into vascular phenotypes^18^. Also, they are more readily available and proliferative for cell sheet formation compared with SMCs^19^. While mesenchymal stem cells (MSCs) do not normally differentiate into SMC phenotypes, co-culture of MSCs with ECs promotes the MSC differentiation towards a more contractile phenotype, and the exposure to flow leads to further enhancement of contractile protein expression in MSC^20^,^21^.

In this study, we fabricated a small-diameter (1 mm i.d.) TEBV comprising hMSCs in the wall and human cord blood derived endothelial progenitor cells (hEPCs) on the luminal surface without any synthetic or exogenous materials. To mimic the 3D spiral and interwoven organization of SMCs in native blood vessels, we prepared aligned hMSC cell sheets on nanogratings and wrapped these cell sheets around a temporary supporting mandrel in a layer-by-layer fashion. Maturation for 2 weeks and removal from the mandrel yielded a tubular structure with circumferentially aligned hMSCs. The lumen size of the tubular structure was controlled by the diameter of the mandrel, 1 mm, and the wall thickness of the TEBVs was determined by the number of cell layers and maturation time. We characterized our scaffold-free TEBV and compared with those obtained from native porcine vessels. Scanning electron microscopy (SEM) and immunofluorescent staining were used to visualize micro-/nanoscale features of TEBV and detect SMC, EC and extracellular matrix protein expression, respectively. The functionality of the TEBV was assessed by vasodilation and vasoconstriction in response to perfusion within the normal physiological range. In addition, the functionality of the endothelium was assessed with respect to nitric oxide (NO) release and monocyte adhesion. Finally, the mechanical strength was evaluated through burst pressure analysis.

## Results

**Figure 1:**
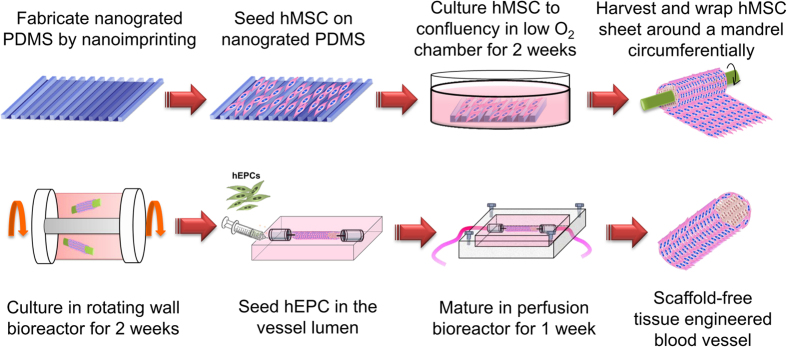
Overall experimental scheme of fabricating an hMSC-based scaffold-free TEBV.

**Figure 2:**
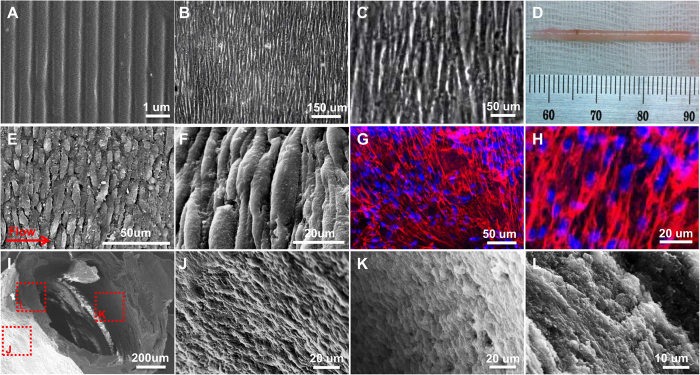
Images of aligned cell sheets and resulting TEBV. (**A**) SEM image of nanopatterned PDMS; (**B,C**) Phase images of aligned hMSCs grown on nanopatterned PDMS, (**D**) hMSC sheets wrapped around 1.3 mm diameter glass rod; (**E,F**) SEM images of circumferentially aligned hMSC in outer layer of TEBV; (**G,H**) Confocal images showing phalloidin staining (Red) and DAPI (Blue) of TEBV; (**I–L**) SEM images of various TEBV sections demonstrating that the cell sheets were well fused to create multiple layers of the vessel wall with confluent cell density throughout the vessel from the lumen to the exterior ((**I**): TEBV; (**J**): longitudinal section showing lumen; (**K**): longitudinal section showing the outer wall; and (**L**): cross-section)

Figure 1 shows the overall experimental scheme for fabricating our scaffold-free TEBV. In our scheme, four layers of aligned cell sheets, which were grown on nanopatterned PDMS mold (Fig. 2A) under hypoxic (2% O_2_) conditions for 3 weeks, were wrapped around a mandrel (Fig. 2D) and cultured for an addition of 2 weeks. We have previously shown that the hypoxic environment helps sustain hMSC viability and multipotency and therefore facilitates the formation of a uniform and confluent hMSC cell sheet^22^. The uniform alignment could be seen in microscopic images of hMSCs grown on nanopatterned PDMS (Fig. 2B,C). The percentage of cell sheet shrinkage caused by the wrapping process was 16.7 ± 3.2% for n = 12. Because this measurement was consistent and therefore predictable, the reproducibility of the TEBV fabrication was not affected.

Once the cell sheets had fused and matured into a tubular vessel form, the mandrel, which acts as a temporary scaffold, was removed. The outer diameter of the TEBV was measured as 1.13 ± 0.44 mm. The scaffold-free TEBV was then placed in a perfusion bioreactor, and hEPCs were seeded into the TEBV lumen. The perfusion bioreactor was set under a continuous flow at 2 mL/min (physiological shear stress of 6.8 dynes/cm^2^) for a week before any analysis was conducted, as previous studies have shown that exposure to flow conditions increase the mechanical strength, contractility and smooth muscle cell differentiation of a TEBV^23^,^24^. The burst strength of matured TEBVs was measured with a specially designed system pressurized with PBS solution. Hydrostatic pressure was increased slowly until sample failure. The burst pressure was measured as 342.3 ± 101.2 mmHg. This burst pressure is higher than that of human physiological vascular microenvironments (normally < 200 mmHg)^25^, suggesting that the TEBV can be used in microphysiological systems representing native human tissue environments.

Immunofluorescence staining of actin filaments showed that the hMSCs were confluent and uniformly aligned in the TEBV after 3 weeks of maturation under perfusion (Fig. 2G,H). In addition, SEM images of various TEBV sections (cross-section, longitudinal section showing the lumen, and longitudinal section showing the outer wall) demonstrated that the cell sheets were well fused to create multiple layers of the vessel wall with confluent cell density throughout the vessel from the lumen to the exterior (Fig. 2I–L). The H&E staining of the TEBV cross section further confirms the maturity of the vessel wall (Supplementary Fig. S1).

**Figure 3:**
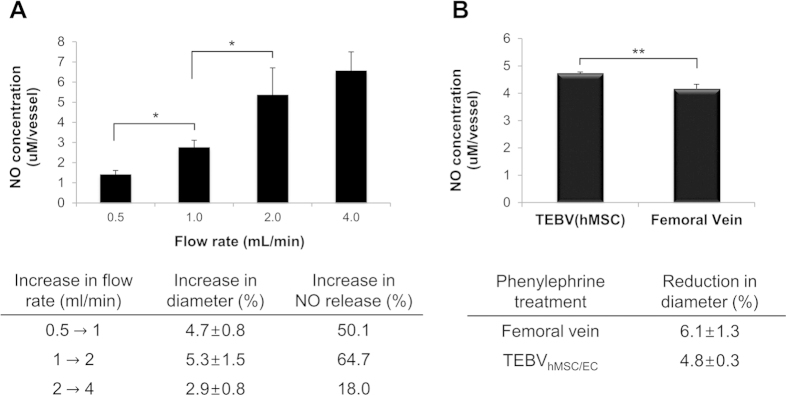
Response of scaffold-free TEBVs to flow rate (**A**) and 1 μM phenylephrine (**B**). The flow rate was gradually increased from 0.5 to 4 mL/min in doubling increments and the vessel diameter increased as the flow rate increased (*p < 0.05). The decrease of vessel diameter and the amount of NO release in response to the drug was comparable to those of native vessels (**p > 0.05).

The functionality of the cell sheet layer of our scaffold-free TEBV was assessed by measuring the vasodilation and vasoconstriction of the vessel in response to flow and to 1 μM phenylephrine, a vasoconstrictor drug, respectively (Fig. 3). To test vasodilation, the flow rate was gradually increased from 0.5 to 4 mL/min in double increments. The vessel diameter increased as the flow rate increased. This increase was distinct enough to be visualized, as evident in the supplementary video S1 online. The percent increase of the vessel diameter at each phase of flow rate increase is shown in Fig. 3. In addition, the amount of nitric oxide (NO) released by the TEBV at each flow rate was measured as another benchmark to determine the functionality of the endothelium^26^. The NO measurements showed a similar trend as that of vasodilation: the NO concentration increased with the flow rate. This result agrees with previous findings that a functional endothelium produces more NO when exposed to fluid shear stress^27^.

To test vasoconstriction, phenylephrine, a vasoconstrictor drug that acts as α-adrenergic receptor agonist for vascular smooth muscle cells, was added to the perfusion bioreactor. Results indicated that 1 μM of phenylephrine treatment reduced the vessel diameter by 4.8 ± 0.3% (Fig. 3). This reduction in diameter was not statistically different than the reduction in diameter observed in native porcine femoral vein under identical treatment (6.1 ± 1.3%). Furthermore, the amount of NO released by the TEBV under phenylephrine treatment was comparable to that released by native porcine femoral vein (p > 0.05).

**Figure 4:**
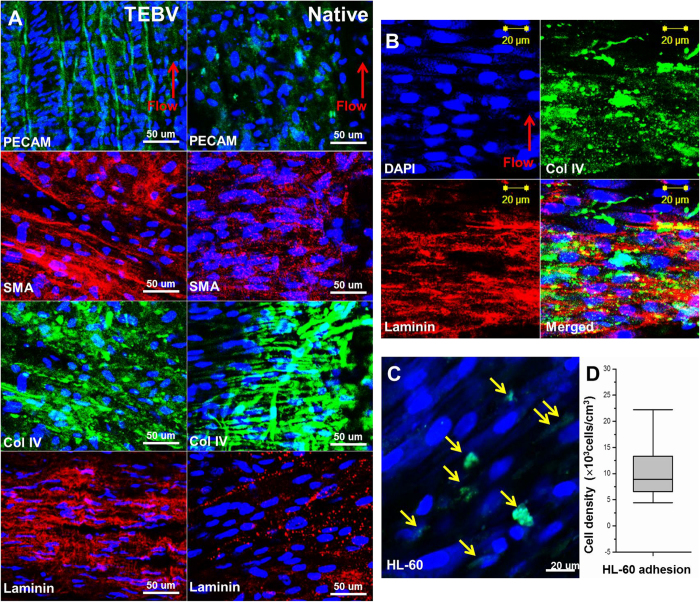
Confocal images showing immunostaining for PECAM, SMA, Type IV collagen, and laminin of TEBV cultured for 7 days in the perfusion chamber and native porcine femoral vein, respectively (**A**).Immunofluorescence staining images show that the hMSCs align perpendicular to the direction of flow in the bioreactor, which is consistent with previous report that smooth muscle cells tend to orient in a direction perpendicular to blood flow (**B**). Monocyte adhesion assay shows the attachment of HL-60 s (arrows) to the endothelial cells and beside the aligned smooth muscle cells in the TEBV (**C**). Density of the attached HL-60 cells on the activated endothelium of TEBV is shown in (**D**). No attached HL-60 cells were observed if the TEBV was not endothelialized. Error bar indicates standard error.

The level of extracellular matrix production, the extent of hMSC differentiation towards the SMC phenotype, and the degree of endothelial coverage of TEBV after 7 days of perfusion was analyzed and compared to those of the native vessel (positive control) (Fig. 4A). Immunofluorescent staining against ECM components in the basal lamina (collagen IV and laminin), SMC (α-smooth muscle actin), and EC (platelet endothelial cell adhesion molecule (PECAM)) markers were performed. The level of each marker expression in the TEBV increased after perfusion (from d0 to d7) (data not shown), and became comparable to that of the native vessel. The increased expression of collagen IV and laminin in Fig. 4 showed that hMSCs in the TEBV wall secrete basal lamina ECM proteins that surround the smooth muscle layer. Furthermore, the high fluorescence intensity shown for SMC and EC markers indicated an MSC-to-SMC differentiation and the presence of a confluent endothelium, respectively. Moreover, the immunofluorescent staining in Fig. 4B showed that hMSCs in the TEBV wall aligned perpendicular to the direction of flow in the bioreactor. On the other hand, the immunofluorescent staining against PECAM showed that endothelial cells aligned parallel to the direction of flow. This observation is consistent with both the staining obtained from the native vessel in Fig. 4A, and previous reports that smooth muscle cells tend to orient in a direction perpendicular to the blood flow^28^, while endothelial cells orient in a direction parallel to the blood flow^29^.

To further test the functionality of the endothelium in the TEBV, monocyte adhesion in the TEBV was measured after TNF-α activation. Figure 4C shows the strong attachment of monocyte-like HL-60 cells on the activated endothelium of the TEBV even after 4 hours of continuous perfusion. The image was taken with the lumen facing up, which indicated that the stained HL-60 attached to the endothelial cells and beside the aligned smooth muscle cells in the TEBV. The attached cell density at different views was measured and shown in Fig. 4D. A relatively good uniformity of endothelium in the TEBV could also be seen. In contrast, monocyte adhesion was not observed in the control TEBV without TNF-α treatment (data not shown).

## Discussion

Microphysiological systems using human cell-based tissues for *in vitro* drug screening and toxicology evaluation may overcome some of the drawbacks associated with rodent animal models due to their potential of eliciting a more human-like response. A TEBV mimicking a native vessel would be an important addition to an integrated multi-organ microphysiological system, which not only serves as a mass transport system for nutrients and cytokines, but also more faithfully recapitulates the *in vivo* situation where drug exposure to the endothelium may obscure the toxic effects in other tissues. Such TEBV can also act as a screening system for drugs to treat vascular diseases such as atherosclerosis. In this study we created a scaffold-free TEBV mimicking native vessel made up of aligned hMSC cell sheets and hEPCs. By removing any exogenous and synthetic materials from the TEBV construction, we hypothesize that such TEBV may have a greater likelihood of achieving acceptable functionality for drug screening^30^,^31^. Work is underway to compare the functionality of this scaffold-free configuration with the one involving dense collagen gel in the construction of TEBV. A scaffold-free configuration holds the advantage of achieving a high cell density in a scaffold-based TEBV^32^. After all, a native blood vessel contains a highly dense SMC layer. Pre-aligning the cells in the cell sheet method may also approximate the 3D spiral and interwoven organization that exists in the medial layer of a native blood vessel.

The cell sheet-based construction loses the ancillary mechanical support of a scaffold, which may be detrimental for *in vivo* applications. This concern is alleviated for drug testing *in vitro*. Nevertheless, the burst pressure obtained for the 3-layered TEBV in this study is within range of that for native human vessel^25^. Moreover, the mechanical properties of the proposed TEBV can be improved by using more cell layers as well as a longer maturation time. However, such gain must be weighed against the materials and time needed to construct the TEBV. On balance, the protocol described in this study appears adequate to produce a construct reasonably functional within five weeks.

The functions of the TEBV were assessed in terms of flow-mediated vasodilation, phenylephrine-induced vasoconstriction, and the corresponding NO release under each stimulus. Flow-mediated vasodilation is a noninvasive diagnosis for detecting endothelial dysfunction in systemic arteries, particularly for patients with risk of atherosclerosis or coronary artery disease^33^,^34^. Studies indicate that an increase in flow rate causes dilation of vessels by releasing endothelium-derived relaxing factor such as NO^35^, and this dilation is dependent on an intact, functional endothelium^26^. In accordance with the previous findings, our results shown in Fig. 3 indicate that increase in flow rate causes an increase in both the vessel diameter and the NO release. This implies that our TEBV has a functional endothelium that modulates vessel tone in response to external stimuli such as flow rate. Indeed, the immunofluorescence staining for PECAM in TEBV indicates the presence of a confluent layer of endothelial cells in the vessel lumen (Fig. 4). In addition, administration of 1 μM of phenylephrine, which acts on vascular smooth muscle cells to create vasoconstriction^36^, showed similar percentage decrease in the vessel diameter between the TEBV and the native porcine femoral vein (Fig. 3). The level of NO release under phenylephrine treatment was also similar. Therefore, it suggests that the TEBV possesses hMSCs that exhibit a contractile SMC phenotype. This was confirmed by the expression of a SMC marker, alpha-smooth muscle actin, in Fig. 4. Furthermore, Lee *et al.* have studied the flow-stimulated perpendicular alignment of SMCs in the vessel wall^28^. They stated that the perpendicular orientation of SMCs is important for the mechanical strength and the function of the blood vessels, and such alignment is regulated by mechanotransduction mechanisms often observed when SMCs are subjected to high strain^28^. Thus, the perpendicular orientation of our hMSCs in the TEBV (Fig. 4B) suggests that the hMSCs exhibit a SMC phenotype and allow for proper vascular function.

The monocyte adhesion assay further validated the presence of a functional endothelium in our TEBV. Inflammation is a crucial nonspecific defense against tissue damage, irritant or pathogen, and the interaction between vascular endothelium and leukocytes is necessary for a complete inflammatory response to develop^37^. Following injury or a pro-inflammatory stimulus, various cytokines, such as TNF-α, induce the up-regulation of adhesion molecule expression on the surface of endothelial cells to initiate the binding of leukocytes and develop inflammatory responses^38^. In this study, we observed the adhesion of monocyte-like HL-60 to the endothelial cells in the TNF-α-activated TEBV lumen, but not to the TEBV without endothelialization. This demonstrates that a functional endothelium is necessary for monocyte adhesion, and that our TEBV contains such mature endothelial layer.

So far our TEBV has only been compared to the native animal vessel. Although the assessments demonstrate comparable functionality, there is a need for direct comparison with native human vessels under the same stimuli. In fact, the goal of our future studies is to test drug compounds that have shown poor correlation between pre-clinical studies and clinical trials, so as to validate the use of TEBV as a drug screening platform. In summary, this study is a first step towards the development of a human cell-based microphysiological system that incorporates drug exposure to an endothelium as an integral part of drug screening.

## Methods

### Fabrication and characterization of PDMS nanogratings substrates and aligned MSC sheets

PDMS substrates with nanogratings of 350 nm line width, 700 nm pitch, and 250 nm depth were used for generating pre-aligned MSC sheets. Briefly, the nanopattern was fabricated on the poly(methyl methacrylate)-coated Si wafer by using electron beam lithography and then replicated on PDMS (Ellsworth Adhesives, Germantown, WI) using soft lithography. To create multiple copies of PDMS substrates, the original PDMS substrate was imprinted onto polystyrene (PS) disks under molding conditions of 210 °C and 50 kPa for 5 min, followed by cooling to room temperature. A mixture of PDMS resin and curing agent (SYLGARD 184 kit, Dow Corning, MI, USA) was poured onto the nanograted PS mold (L = 6 cm, W = 4.5 cm) and cured for 2 h at 65 °C. Before culturing MSC on the PDMS substrates, the substrates were oxygen-plasma treated with a Trion Phantom II Reactive Ion Etcher for 60 s, 300 mTorr chamber pressure, 20 cm^3^ O_2_ flow rate, and 20 W RF power. Plasma-treated substrates were immediately immersed in sterile EtOH for sterilization, and then coated with rat tail collagen I (Sigma-Aldrich, Germany) at 15 mg/cm^2^ for 1 h to facilitate hMSC attachment^22^,^39^,^40^.

Bone marrow–derived hMSCs were generously provided by Darwin J. Prockop of Texas A&M Institute for Regenerative Medicine. hMSCs below passage 5 were seeded at 5,000 cells/cm^2^ on collage coated, nanopatterned PDMS substrates and cultured using complete media [α-minimum essential medium (Gibco) with 20% fetal bovine serum (Atlanta Biologicals), 1% penicillin/streptomycin (Life Technologies, Rockville, MD), and 82.5 μg/ml L-Ascorbic acid (Sigma-Aldrich)] at 37 °C in a hypoxic chamber of 2% O_2_ for 3 weeks.

The pattern transfer fidelity from mold to replica and cell alignment onto the PDMS substrates were examined using scanning electron microscopy (SEM) (FEI XL30 SEM-FEG). Samples, either only the PDMS substrate or the PDMS substrate with hMSCs seeded, were fixed, dehydrated and dried, and then coated with a 10 nm layer of gold film (Denton Desk IV vacuum sputter coater) to improve sample conductivity for SEM imaging.

### Fabrication of functional scaffold-free TEBVs

To create a tubular vessel structure using hMSC sheets, confluent hMSC layers were carefully detached from the PDMS with tweezers and wrapped onto a temporary supporting mandrel (glass rod, 1 mm diameter). Typically four hMSC sheets were rolled around a mandrel to form a TEBV. These TEBVs were then stabilized in a complete media inside hypoxic chamber overnight to allow the cells to fuse together. For further maturation of the cellular assemblies, the cell sheets with the mandrel were cultured in a rotating wall bioreactor (Synthecon, TX) at 37 °C in the hypoxic incubator of 2% O_2_ for 2 weeks. Media was replaced every 5–7 days. The temporary mandrel was then removed from the TEBV with tweezers, and the TEBV was inserted into a perfusion chamber (KISTECH, Korea).

Human umbilical cord blood derived endothelial progenitor cells (hEPCs) were isolated as previously described^41^. Umbilical cord blood was obtained from the Carolina Cord Blood Bank. All patient identifiers were removed prior to receipt. The protocol for the collection and the usage of human blood in this study was approved by the Duke University Institutional Review Board.

hEPCs (5 × 10^5^ cells/vessel of 1 mm i.d. and 3 cm length) were seeded into the vessel by injection into the chamber using a 3 mL syringe with Luer-Lok^TM^ Tip (BD Biosciences), and the chamber was rotated for 30 min with 0.17 rpm in the incubator to allow for an even attachment of endothelial progenitor cells around the vessel lumen. The TEBV was then connected to a perfusion chamber running at a steady flow of 2 mL/min and further cultured for 2 weeks in 37 °C and 5% CO_2_^21^. The overall experimental scheme is shown in Fig. 1.

### Characterization of scaffold-free TEBV

To examine the mechanical property of TEBV, burst pressure strength was measured. TEBVs were mounted in a system custom-designed for pressurizing individual vessels until failure. TEBVs were pressurized with water at increments of 300 mmHg in approximately 2 minutes until rupture.

The biological activity of TEBVs was evaluated after 14 days of culture in perfusion chambers. To examine the change in the diameter of the TEBV in response to flow-mediated vasodilation and a vasoconstrictor drug, phenylephrine, the perfusion chamber connected with TEBVs was placed on a microscope stage (SM3-TZ, Amscope, CA) equipped with a video camera and computer with ImageJ software. The chamber was perfused with a culture media at the start of the recording. Vessel diameters were measured at various flow rates, and before and after perfusion with the vasoconstrictor phenylephrine (1 μM). Measurements were typically taken 5 min after perturbation. Media samples were collected to measure nitric oxide (NO) release. Media samples were frozen at −80 °C and lyophilized (Appropriate Technical Resources, Laurel, MD) for 24 h. The lyophilized medium was resuspended into 1/10 of its original volume in double-distilled water (ddH_2_O), and mixed with equal volumes of 1X Greiss Reagent (Sigma) in a 96 well plate (BMG Labtech, Ortenberg, Germany). After 15 min, spectrophotometric absorbance was measured at 540 nm. A standard curve was prepared using sodium nitrite solutions (Sigma) ranging from 0 to 80 μM in ddH_2_O. The values were analyzed by one-way analysis of variance (ANOVA), followed by nonparametric LSD tests. A p < 0.05 was considered to indicate statistical significance.

To observe the distribution of vascular extracellular matrix and cellular alignment of matured vessels, immunofluorescence staining was performed. TEBVs were fixed with 4% parafomaldehyde (Electron Microscopy Sciences) in PBS for 24 h at room temperature, and permeabilized in a blocking solution consisting of 0.03 g/ml bovine serum albumin (BSA, Sigma) and 0.1% goat serum (Sigma) dissolved in 0.2% Triton X-100 (Sigma) diluted with PBS for 2 h. Then, TEBVs were stained with platelet endothelial cell adhesion molecule (PECAM, Abcam, Cambridge, MA), alpha-smooth muscle actin (α-SMA, Abcam, Cambridge, MA), Type IV collagen (Abcam, Cambridge, MA) and laminin (Abcam, Cambridge, MA) antibodies. Different Alexa Fluor secondary antibodies (Abcam, Cambridge, MA) were used to obtain fluorescent colors. Also, the cell nuclei were stained with 4, 6-diamidino-2-phenylindole (DAPI, Molecular Probes). After the staining, the samples were then mounted in Fluoro-Gel (Electron Microscopy Sciences, Hatfield, PA) and viewed with a Zeiss LSM 510 inverted confocal microscope.

To test the functionality of the endothelium in TEBV, monocytes adhesion onto the activated endothelium was measured. First, HL-60 monocyte-like cells (CCL-240; American Type Culture Collection, Rockville, MD) were labeled with 2 μM CMFDA (Molecular Probes) in culture medium for 30 minutes. The TEBV was labeled with 0.5 μM DAPI (Molecular Probes) after fixation and permeabilization. For endothelial activation measurement, the TEBV was perfused with culture medium containing 500 U TNF-alpha for 4 hours, and then monocyte-like HL-60 cells (10^5^ cells/mL) were perfused through the TEBV at a shear stress of 1.4 dynes (perfusion speed at 2 mL/min) for an additional 40 min. The TEBV was gently washed with medium without HL-60 cells three times and then fixed with 4% PFA in PBS. The TEBV was then cut open and imaged on the lumen side using a Zeiss LSM 510 inverted confocal microscope. For HL-60 cell counting, the fluorescent images showing labeled HL-60 cells were analyzed using Image J software.

### Ethics Statement

All methods used in this study were carried out in accordance with the approved ethical guidelines of all institutions. The study protocol was approved by the Duke University Institutional Review Board.

## Additional Information

**How to cite this article**: Jung, Y. *et al.* Scaffold-free, Human Mesenchymal Stem Cell-Based Tissue Engineered Blood Vessels. *Sci. Rep.* **5**, 15116; doi: 10.1038/srep15116 (2015).

## Supplementary Material

Supplementary Video S1

Supplementary Information
