# Supplementary material for: Scaffold-free, Human Mesenchymal Stem Cell-Based Tissue Engineered Blood Vessels
Source: Sci Rep. 2015 Oct 12;5:15116. doi: 10.1038/srep15116 (PMC4600980; doi:10.1038/srep15116)
Supplement: Supplementary Information [file srep15116-s2.pdf]

Supplementary Information

Scaffold-free, Human Mesenchymal Stem Cell-Based Tissue Engineered Blood Vessels

Youngmee Jung, HaYeun Ji, Zaozao Chen, Hon Fai Chan, Leigh Atchison, Bruce Klitzman, George Truskey and Kam W. Leong

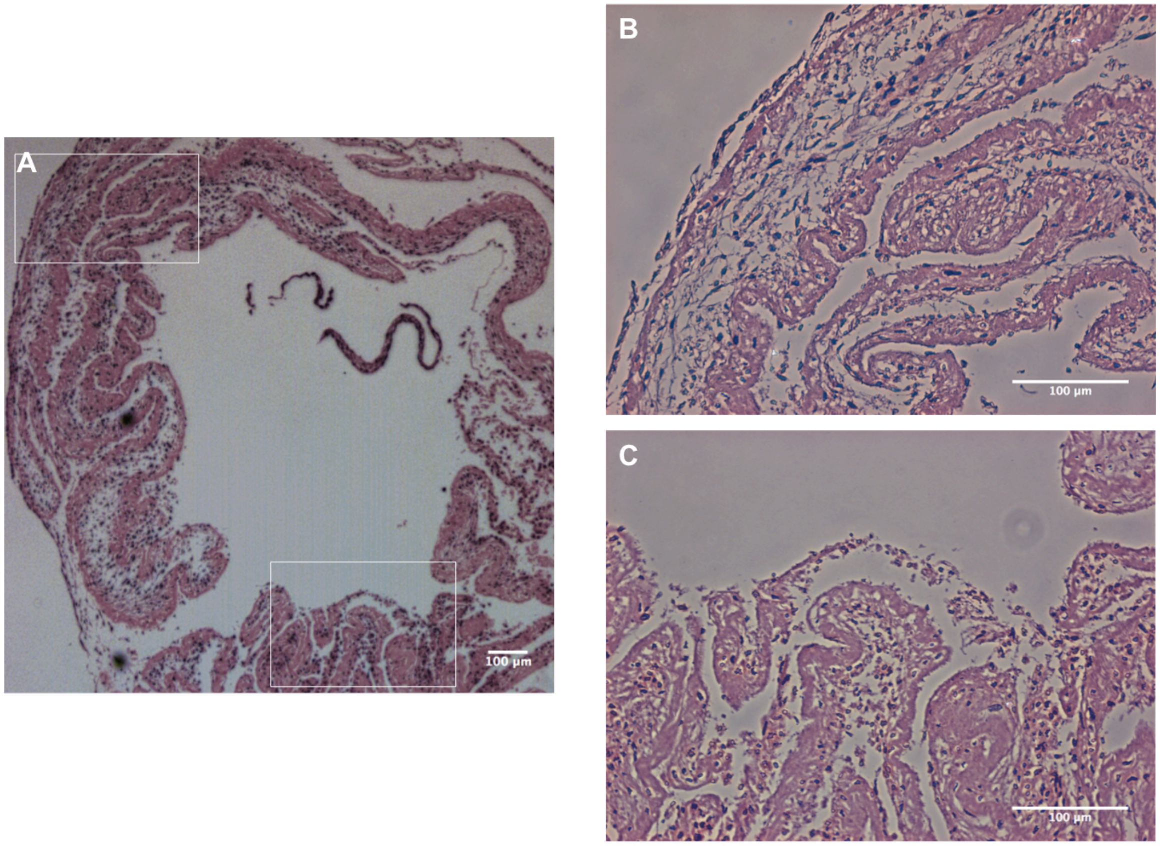

Supplementary Figure S1. Histological cross section of the scaffold-free TEBV stained with hematoxylin and eosin (H&E). (A) Low magnification image showing the whole cross-sectional view of the TEBV, (B, C) Magnified images of sections of the TEBV indicated by the boxes in (A).
